# *C1QA*, *C1QB*, and *GZMB* are novel prognostic biomarkers of skin cutaneous melanoma relating tumor microenvironment

**DOI:** 10.1038/s41598-022-24353-9

**Published:** 2022-11-28

**Authors:** Zhuoshuai Liang, Lingfeng Pan, Jikang Shi, Lianbo Zhang

**Affiliations:** 1 Department of Epidemiology and Biostatistics, School of Public Health, Jilin University, Changchun, 130021 Jilin China; 2 Department of Plastic Surgery, China Japan Union Hospital of Jilin University, Changchun, 130033 Jilin China

**Keywords:** Functional genomics, Diagnostic markers, Predictive markers, Biomarkers

## Abstract

Skin cutaneous melanoma (SKCM) is the most lethal form of skin cancers owing to high invasiveness and high metastatic potential. Tumor microenvironment (TME) provides powerful evidences for discerning SKCM, raising the prospect to identify biomarkers of SKCM. Based on the transcriptome profiles of patients with SKCM and the corresponding clinical information from The Cancer Genome Atlas (TCGA), we used ESTIMATE algorithm to calculate ImmuneScore and StromalScore and identified the TME-Related differentially expressed genes (DEGs), than the intersected TME-Related DEGs were used for subsequent functional enrichment analysis. Protein–protein interaction (PPI) analysis was used to identify the functionality-related DEGs and univariate Cox regression analysis was used to identify the survival-related DEGs. Furthermore, SKCM-related DEGs were identified based on two Gene Expression Omnibus (GEO) datasets. Finally, we intersected functionality-related DEGs, survival-related DEGs, and SKCM-related DEGs, ascertaining that six DEGs (*CCL4*, *CXCL10*, *CCL5*, *GZMB*, *C1QA*, and *C1QB*) function as core TME-related genes (CTRGs). Significant differences of *GZMB*, *C1QA*, and *C1QB* expressions were found in gender and clinicopathologic staging of SKCM. High levels of *GZMB*, *C1QA*, and *C1QB* expressions were associated with favorable prognosis. Gene set enrichment analysis (GSEA) showed that cell–cell interaction, cell behavior, and intracellular signaling transduction may be mainly involved in both *C1QA*, *C1QB* and *GZMB* expressions and metabolism of phospholipid and amino acid, transcription, and translation may be implicated in low *GZMB* expressions. *C1QA*, *C1QB*, and *GZMB* are novel SKCM-relating CTRGs, providing promising immune-related prognostic biomarkers for SKCM.

## Introduction

Skin cutaneous melanoma (SKCM), the most lethal form of skin cancers, causes 75% of skin cancer deaths owing to high invasiveness and high metastatic potential^1^. For most early-stage SKCM, surgical resection is an optimal treatment, but effective therapies are limited for late-stage SKCM^2^. However, naked eyes or dermatoscopes are difficult to directly distinguish SKCM from melanocytic nevus, and many patients have experienced invasion and metastasis when they are diagnosed as SKCM^3,4^. Notably, tumor microenvironment (TME) provides powerful evidences for discerning SKCM, raising the prospect to identify biomarkers of SKCM^5^.

The infiltration of multiple immune cell subsets is involved in TME. TME is critical for tumor invasion, angiogenesis, unlimited proliferation and even immune escape^5,6^. High levels of immune-cell infiltration correlate with favorable prognosis, suggesting that the tumor microenvironment-related ImmuneScore and the proportions of tumor-infiltrating immune cells (TICs) in the TME may provide a new dimension for predicting and treating cancers, such as SKCM^7–10^. Heterogeneous immune cells and stromal cells confer the basis of ImmuneScore and TME^9,11^, providing direct evidence on the prognostic biomarkers of SKCM, including *CXCL9*, *CXCL10*, *CXCL13*, *CCL4*, and *CCL5*^10^*.* In this present study, we performed an integrated bioinformatic analysis on the basis of multiple databases using bioinformatic algorithms and tools, identifying that *GZMB*, *C1QA*, and *C1QB*, as immune-related biomarkers for SKCM, can be feasible for predicting prognosis and immunotherapy efficacy. We present the following article in accordance with the STROBE reporting checklist.

## Materials and methods

### Data sources

The transcriptome profiles of patients with SKCM and the corresponding clinical information were downloaded from the Cancer Genome Atlas (TCGA) (https://portal.gdc.cancer.gov/), the Genotype-Tissue Expression (GTEx) datasets (https://toil-xena-hub.s3.us-east-1.amazonaws.com/download/GTEX_phenotype.gz), and Gene Expression Omnibus (GEO) datasets (https://www.ncbi.nlm.nih.gov/geo/).

To minimize potential batch effects, same library preparation and sequencing platform was used to analyze the gene expressions data from TCGA and GTEx (https://toil-xena-hub.s3.us-east-1.amazonaws.com/download/gtex_RSEM_gene_fpkm.gz). After merging these two datasets, the data of 471 SKCM samples from TCGA and 813 non-SKCM samples of skin from TCGA and GTEx were finally selected. GSE46517 (104 SKCM samples and 17 non-SKCM samples of skin) and GSE15605 (58 SKCM samples and 16 non-SKCM samples of skin) from GEO were used to identify differentially expressed genes (DEGs) respectively. In addition, GSE65904 and GSE54467 were used to verify the predictive effect of DEGs on survival of patients with SKCM.

### Identification of DEGs between SKCM and non-SKCM

Integration of multiple arrays is considered a better approach of enhancing the reliability of results than individual array analysis^12^. We used GEO2R (http://www.ncbi.nlm.nih.gov/geo/geo2r) to identify DEGs between SKCM samples and non-SKCM skin samples from the two GEO Datasets (GSE46517 and GSE15605) on the basis of the threshold of |log2(fold change)| > 1 and FDR-adjusted *P* < 0.01.

### Associations of ImmuneScores, StromalScores, and ESTIMATEScores with survival and with features of patients with SKCM

ImmuneScore, StromalScore, and ESTIMATEScore have been widely used: the ImmuneScore, an index reflecting proportions of immune components, is defined to describe the infiltration of immune cells in tumour tissue; the StromalScore, an index reflecting proportions of stromal components, is defined to represent the presence of stroma in tumour tissue; and the ESTIMATEScore, an index reflecting TME, is defined as the sum of the ImmuneScore and the StromalScore^7–9,13^. The ImmuneScore, StromalScore, and ESTIMATEScore were calculated using the ESTIMATE algorithm through “ESTIMATE” R package^13^. We divided the 471 SKCM samples from TCGA into two corresponding groups according to the medians of the ImmuneScores, StromalScores, and ESTIMATEScores, respectively. Kaplan–Meier survival curves were performed through “Survival” and “survminer” R packages to evaluate overall survival (OS). OS was compared using the log-rank test, with *P* < 0.05 being considered significant. Additionally, Kruskal–Wallis rank sum test was performed using “ggpubr” R package assess associations of the above scores with age, gender, T stages (T0, T1, T2, T3, T4, and Tis), N stages (N0, N1, N2, and N3), M stages (M0 and M1), overall stage, or tumor metastasis, respectively.

### Identification of TME-related DEGs

Patients with SKCM on the basis of the medians of the ImmuneScores and StromalScores, were divided into two immune groups (high-immune-score group and low-immune-score group) and two stromal groups (high-stromal-score group and low-stromal-score group), respectively. For the two immune groups and the two stromal groups, DEGs were identified using the “limma” R package. False discovery rate (FDR)-adjusted *P* < 0.001 and |log2(fold change)| > 1.5 were set as the criteria to screen for DEGs, and results were plotted in volcano plots using the “ggplot2” R package.

### Functional enrichment analysis

To understand the potential biological significance of TME-related DEGs, the Gene Ontology (GO) functional enrichment and the Kyoto Encyclopedia of Genes and Genomes (KEGG) pathway enrichment analyses were performed using the “clusterProfiler”, “enrichplot”, and “ggplot2” R packages (*p* < 0.05, *q* < 0.05)^14,15^. The functional enrichment was visualized using bubble diagram.

Consensus TME-related DEGs confer the contribution to functional enrichment pathways, highlighting the interactions among proteins encoded by TME-related DEGs provide more informative understanding of this contribution^16^. Thus, we analyzed consensus genes and constructed protein–protein interaction (PPI) network using the search tool for the retrieval of interaction genes (STRING) (https://string-db.org/): DEGs with interaction scores > 0.99 were selected for PPI network construction, and PPI network was visualized using Cytoscape v3.8.2. According to the studies of Le et al*.*^17^ and Chen et al*.*^8^, we also selected the top 30 significant TME-related genes using the multi-network clustering (MNC) method through the cytoHubba app in Cytoscape.

### Single-cell analysis

Single-cell analysis was applied using the Tumor Immune Single-cell Hub (TISCH) web tool (http://tisch.comp-genomics.org/documentation/)^18^. The analysis parameters were as follows: C1QA/C1QB/C1QC (Gene), major lineage (Cell-type annotation), and all cancers (Cancer type).

### Identification of core TME-related genes

Univariate Cox regression analysis was performed to identify DEGs that associated with OS using the “survival” R package. *P* < 0.05 indicated statistical significance. In this paper, we selected *P* < 0.001 as a screened criterion to obtain DEGs with much more significance. The association between DEGs and OS was visualized using forest plot. We, further, intersected genes among top 30 significant TME-related genes from PPI, UCRA-derived DEGs with much more significance, DEGs identified from GSE15605, and DEGs identified from GSE46517, focusing on the core TME-related genes (CTRGs) that were finally utilized in differential expressions analysis on combining data between TCGA and GTEx, association analyses of survival and clinicopathological characteristics, and gene set enrichment analysis (GSEA).

For differential expressions analysis on combined data between TCGA and GTEx, CTRGs expressions in SKCM samples were compared with those in non-SKCM samples from TCGA and GTEx using the Wilcoxon rank sum test through the “beeswarm” and “ggpubr” R packages.

For association analyses of survival and clinicopathological characteristics, Kaplan–Meier survival curves were performed through “Survival” and “survminer” R packages to evaluate OS. OS was compared using the log-rank test, with *P* < 0.05 being considered significant. Additionally, Kruskal–Wallis rank sum test was performed using “ggpubr” R package assess associations of CTRGs expressions with age, gender, TNM staging, overall stage, or tumor metastasis, respectively.

For GSEA, the main biological pathways were identified using GSEA v4.1.0 software.

## Results

### Analysis workflow of this study

**Figure 1:**
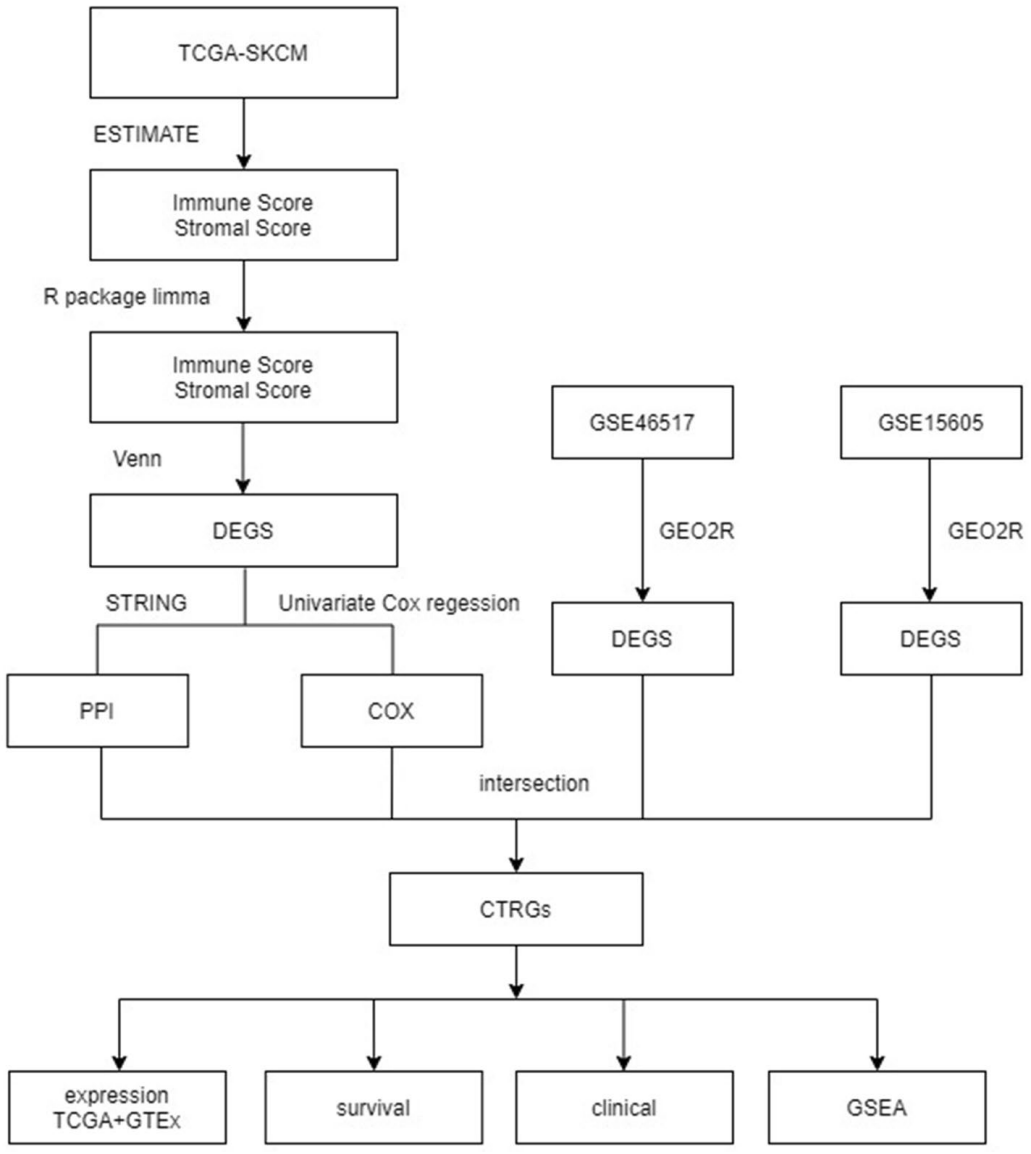
Flowchart of this study.

In this study, we investigated the potential roles and prognostic value of CTRGs in SKCM, as shown in Fig. 1.

### Associations of ImmuneScore, StromalScore, and ESTIMATEScore with survival and clinicopathologic features in SKCM

**Figure 2:**
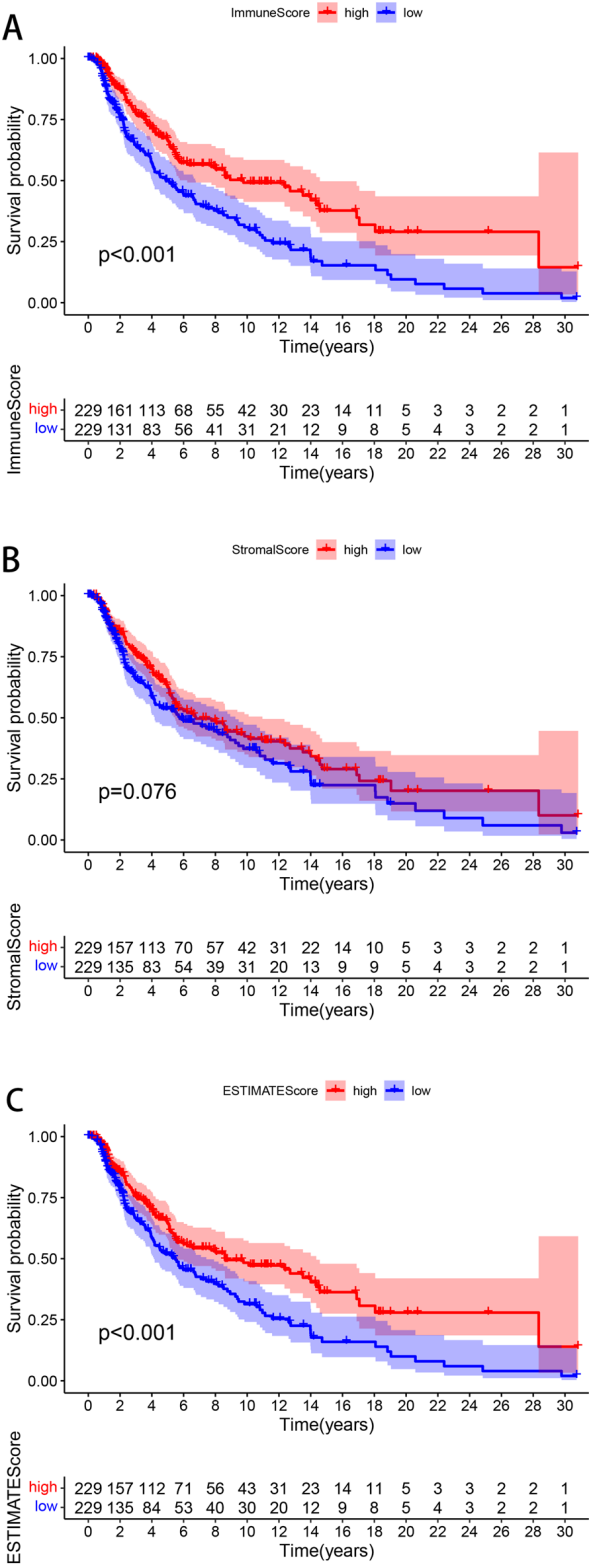
Associations of ImmuneScore, StromalScore, and ESTIMATEScore with survival among patients with SKCM. Kaplan–Meier survival analyses of patients with SKCM with low and high (**A**) ImmuneScores. (**B**) StromalScores. and (**C**) ESTIMATEScores. This figure was generated using “Survival” and “survminer” R packages.

We found that the patients with high ImmuneScore and high ESTIMATEScore exhibited better OS than those with low corresponding scores (Fig. 2A,C), although StromalScore was not significantly associated with OS (Fig. 2B), documenting that the high ImmuneScore are potentially positive prognostic indicators for the patients with SKCM.

**Figure 3:**
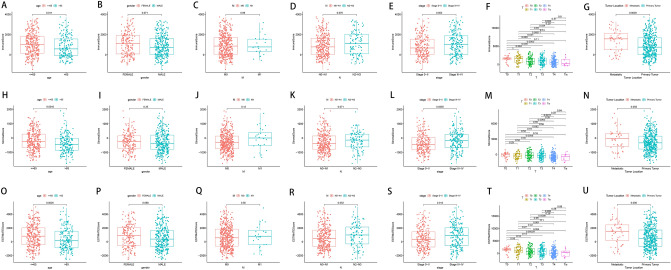
Associations of ImmuneScore, StromalScore, and ESTIMATEScore with clinicopathologic features. Kruskal–Wallis rank sum tests of the associations of (**A**–**G**) ImmuneScore, (**H**–**N**) StromalScore, and (**O**–**U**) ESTIMATEScore with clinicopathologic features. This figure was generated using “ggplot2” R packages.

We, further, analyzed associations of ImmuneScore, StromalScore, and ESTIMATEScore with clinicopathologic features in the patients with SKCM from the TCGA, including age, gender, primary tumor size (T stage), regional lymph node status (N stage), distant metastasis (M stage), overall stage, and tumor metastatic. Patients aged ≤ 65 years had significantly higher scores than patients over the age of 65 (for ImmuneScore, *P* = 0.011; for StromalScore, *P* = 0.0016; for ESTIMATEScore, *P* = 0.0026) (Fig. 3A,H,O). No significant differences of the three scores was found between male and female patients with SKCM (Fig. 3B,I,P), between M stages (M0 and M1) (Fig. 3C,J,Q), and N0–N1 vs. N2–N3 (Fig. 3D,K,R). Notably, the three scores in T4 were significantly lower than those in those in T0, T1, T2, and T3 (all *P* < 0.05) (Fig. 3F,M,T). Overall stage was usually investigated by comparing stage 0–II and stage III–IV^19,20^; thus, we compared the three scores on the basis of stage 0–II and stage III–IV. Interestingly, the StromalScore and ESTIMATEScore in stage III–IV were higher in those in stage 0–II significantly (*P* = 0.0055 and *P* = 0.013, respectively) (Fig. 3E,L,S). Additionally, the ImmuneScore and ESTIMATEScore were significantly higher in metastatic tumors than those in primary tumors (*P* = 0.0039 and *P* = 0.006, respectively) (Fig. 3G,N,U), signifying that the TME may be involved in biological behaviour of SKCM, such as metastasis and infiltration.

### Identification of DEGs reflecting both the high vs. low ImmuneScore and the high vs*.* low StromalScore

**Figure 4:**
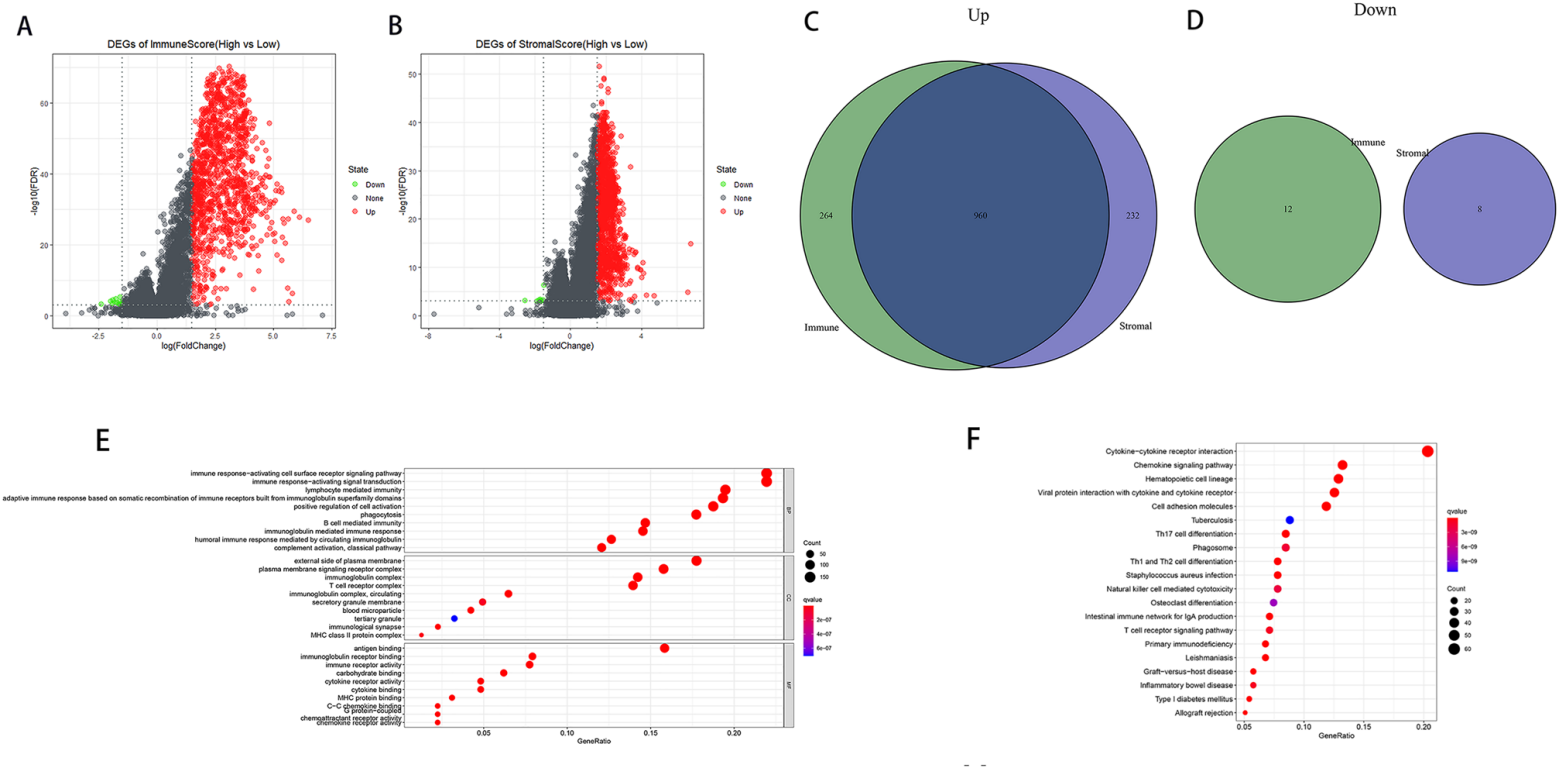
Volcano plots, Venn diagrams, and GO and KEGG enrichment analyses of DEGs. Volcano plots of significant DEGs (FDR-adjusted *P* < 0.001, |log2(fold change)| > 1.5) between high and low (**A**) ImmuneScore and (**B**) StromalScore groups. (**C**,**D**) Venn diagrams of upregulated and downregulated DEGs that were shared by the ImmuneScore and StromalScore analyses. (**E**) GO and (**F**) KEGG enrichment analyses (p < 0.05 and q < 0.05). This figure was generated using “limma”, “ggplot2”, “clusterProfiler”, and “enrichplot” R packages. We have got permission to use the KEGG software from the Kanehisa laboratory (http://www.kegg.jp/kegg/kegg1.html)^15^.

We found 1236 DEGs (1224 upregulated and 12 downregulated) specific to the high vs. low ImmuneScore and 1200 DEGs (1192 upregulated and 8 downregulated) specific to the high vs. low StromalScore groups (Fig. 4A,B). After intersecting these DEGs, we identified 960 upregulated and zero downregulated genes (Fig. 4C,D). We, further, performed GO analysis, unveiling that the 960 DEGs were clustered in the immune-related GO terms, such as immune response–activating cell surface receptor signaling pathway, immune response—activating signal transduction, and B cell mediated immunity (Fig. 4E). Moreover, we uncovered that the DEGs enriched in cytokine–cytokine receptor interaction, chemokine signaling pathway, and hematopoietic cell lineage on the basis of KEGG analysis (Fig. 4F). Thus, both GO and KEGG analyses documented that the 960 DEGs were mainly implicated in immune activity, suggesting that these DEGs may be necessary for the TME of SKCM.

### Confirmation of CTRGs

**Figure 5:**
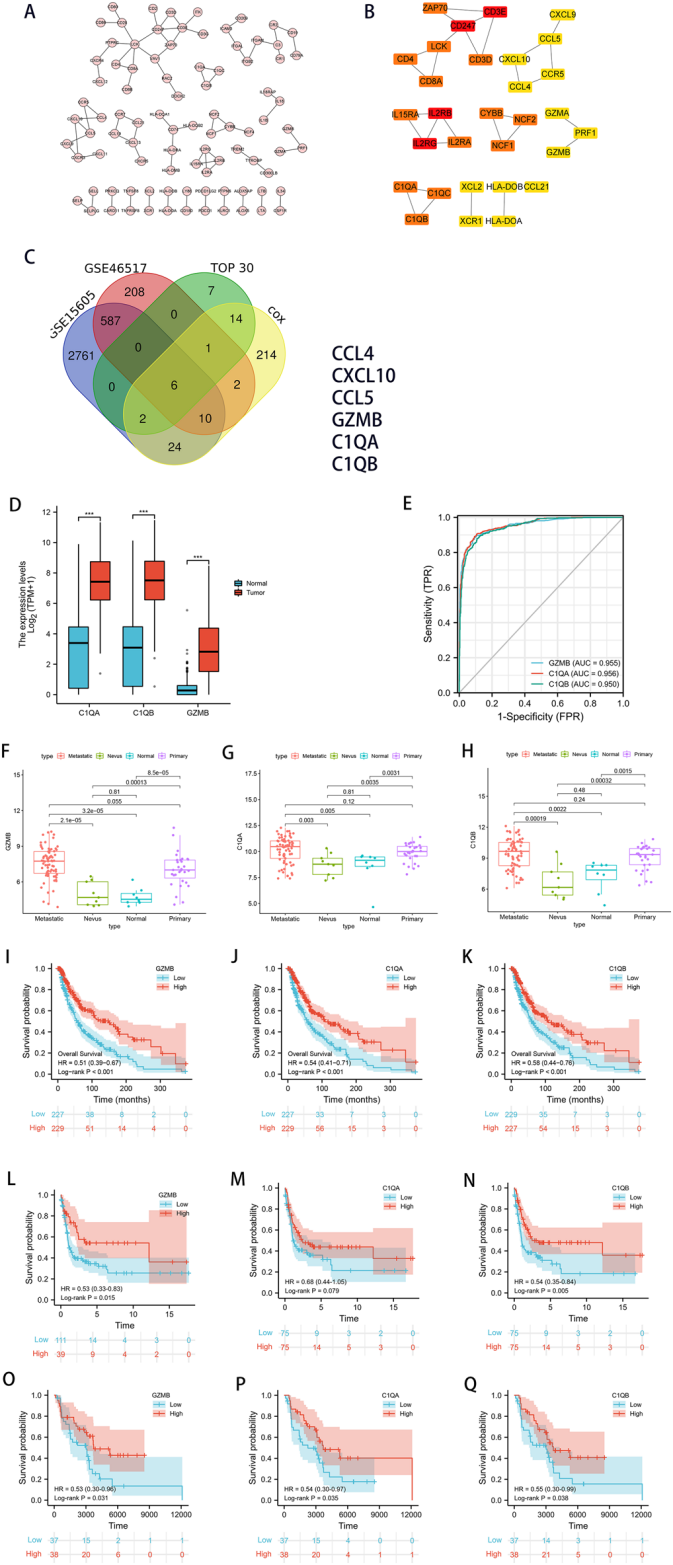
Visualization of the PPI network and correlation of expressions of *GZMB, C1QA,* and *C1QB* with clinicopathologic features. (**A**) PPI network based on STRING confidence score > 0.99. (**B**) Identification of top 30 hub genes in the PPI network using the MNC algorithm. MNC scores increased from yellow to red. (**C**) Venn diagram of the intersection among the functionality-related DEGs, survival-related DEGs, and SKCM-related DEGs. (**D**,**F**–**H**) Comparisons of *GZMB, C1QA,* and *C1QB* expressions levels in SKCM samples and non- SKCM samples. (**E**) AUCs for *GZMB, C1QA,* and *C1QB* expressions. Kaplan–Meier survival curves of *GZMB, C1QA,* and *C1QB* in (**I**–**K**) TCGA, (L-N) GSE65904 and (**O**–**Q**) GSE54467. This figure was generated using the STRING web tool (https://string-db.org/), Cytoscape version 3.8.2, and “beeswarm”, “ggpubr”, “Survival”, and “survminer” R packages.

Firstly, because PPI network analysis frequently used to predict the functionality of interacting genes or proteins reveals the interactions among the genes and proteins^16^, we constructed PPI network according to STRING PPI confidence scores > 0.99, obtaining 90 nodes and 79 edges (Fig. 5A). Moreover, we chose the top 30 hub genes through the MNC algorithm to identify functionality-related DEGs (Fig. 5B). Secondly, we established the association between the 960 DEGs and survival of patients with SKCM using univariate Cox regression analysis, further screening 273 survival-related DEGs (*P* < 0.001) (Fig. S1). Thirdly, to improve detection power, we integrated multiple individual data (2761 DEGs in GSE15605 dataset and 208 DEGs in GSE46517 dataset), confirming SKCM-related DEGs. Finally, we intersected functionality-related DEGs, survival-related DEGs, and SKCM-related DEGs, ascertaining that six DEGs (*CCL4*, *CXCL10*, *CCL5*, *GZMB*, *C1QA*, and *C1QB*) function as CTRGs (Fig. 5C).

### Correlation of expressions of *GZMB, C1QA,* and *C1QB* with survival and with clinicopathological characteristics

Expressions of *GZMB*, *C1QA*, and *C1QB* were significantly lower in SKCM samples than those in non-SKCM samples from TCGA dataset (all *P* < 0.001) (Fig. 5D).

Surprisingly, *GZMB*, *C1QA*, and *C1QB* showed a strong ability to distinguish normal samples from SKCM samples (Fig. 5E). Compared to nevus and normal samples, significant higher expressions of *GZMB*, *C1QA*, and *C1QB* were observed in primary melanoma and metastatic melanoma in GSE46517 (Fig. 5F–H).

Interestingly, patients with SKCM with higher medians of expressions of *C1QA*, *C1QB*, and *GZMB* all had significantly longer OS than those with lower medians in TCGA (Fig. 5I–K) and GSE54467 (Fig. 5O–Q). Moreover, patients with SKCM with higher medians of expressions of *C1QA*, *C1QB*, and *GZMB* all had significantly longer distant metastasis free survival in GSE65904 (Fig. 5L–N).

Additionally, significant differences of *GZMB* expressions were found in gender, and melanoma Clark level (*P* < 0.05) (Table 1). And significant differences of *C1QA* and *C1QB* expressions were found in T stage, pathologic stage, melanoma ulceration, melanoma Clark level, and Breslow depth (*P* < 0.05) (Tables 2 and 3).

**Table 1:** GZMB expressions in clinical features.

Characteristic	Low expression of GZMB	High expression of GZMB	p
n	235	236	
**Gender, n (%)**			0.015
Female	76 (16.1%)	103 (21.9%)	
Male	159 (33.8%)	133 (28.2%)	
**Age, n (%)**			0.678
≤ 60	123 (26.6%)	129 (27.9%)	
> 60	108 (23.3%)	103 (22.2%)	
**Race, n (%)**			0.114
Asian	9 (2%)	3 (0.7%)	
Black or African American	0 (0%)	1 (0.2%)	
White	221 (47.9%)	227 (49.2%)	
**T stage, n (%)**			0.065
T1	14 (3.8%)	27 (7.4%)	
T2	39 (10.7%)	40 (11%)	
T3	50 (13.7%)	41 (11.3%)	
T4	87 (23.9%)	66 (18.1%)	
**N stage, n (%)**			0.587
N0	117 (28.3%)	118 (28.5%)	
N1	41 (9.9%)	33 (8%)	
N2	21 (5.1%)	28 (6.8%)	
N3	27 (6.5%)	29 (7%)	
**M stage, n (%)**			0.204
M0	204 (46%)	214 (48.3%)	
M1	16 (3.6%)	9 (2%)	
**Pathologic stage, n (%)**			0.109
Stage I	33 (8%)	44 (10.7%)	
Stage II	80 (19.4%)	60 (14.6%)	
Stage III	82 (19.9%)	89 (21.6%)	
Stage IV	15 (3.6%)	9 (2.2%)	
**Tumor tissue site, n (%)**			0.750
Extremities	105 (25.1%)	92 (22%)	
Trunk	83 (19.8%)	88 (21%)	
Head and Neck	21 (5%)	17 (4.1%)	
Other	6 (1.4%)	7 (1.7%)	
**Melanoma ulceration, n (%)**			0.198
No	72 (22.9%)	75 (23.9%)	
Yes	95 (30.3%)	72 (22.9%)	
**Melanoma Clark level, n (%)**			0.003
I	4 (1.2%)	2 (0.6%)	
II	8 (2.5%)	10 (3.1%)	
III	26 (8.1%)	51 (15.8%)	
IV	99 (30.7%)	69 (21.4%)	
V	32 (9.9%)	21 (6.5%)	
**Breslow depth, n (%)**			0.133
≤ 3	89 (24.7%)	96 (26.7%)	
> 3	99 (27.5%)	76 (21.1%)	
Age, median (IQR)	59 (48, 70.5)	58 (48, 71)	0.643

**Table 2:** C1QA expressions in clinical features.

Characteristic	Low expression of C1QA	High expression of C1QA	p
n	235	236	
**Gender, n (%)**			0.138
Female	81 (17.2%)	98 (20.8%)	
Male	154 (32.7%)	138 (29.3%)	
**Age, n (%)**			0.547
≤ 60	122 (26.3%)	130 (28.1%)	
> 60	109 (23.5%)	102 (22%)	
**Race, n (%)**			0.320
Asian	8 (1.7%)	4 (0.9%)	
Black or African American	0 (0%)	1 (0.2%)	
White	222 (48.2%)	226 (49%)	
**T stage, n (%)**			< 0.001
T1	12 (3.3%)	29 (8%)	
T2	42 (11.5%)	37 (10.2%)	
T3	43 (11.8%)	48 (13.2%)	
T4	97 (26.6%)	56 (15.4%)	
**N stage, n (%)**			0.311
N0	126 (30.4%)	109 (26.3%)	
N1	36 (8.7%)	38 (9.2%)	
N2	22 (5.3%)	27 (6.5%)	
N3	23 (5.6%)	33 (8%)	
**M stage, n (%)**			0.417
M0	211 (47.6%)	207 (46.7%)	
M1	10 (2.3%)	15 (3.4%)	
**Pathologic stage, n (%)**			< 0.001
Stage I	29 (7%)	48 (11.7%)	
Stage II	90 (21.8%)	50 (12.1%)	
Stage III	78 (18.9%)	93 (22.6%)	
Stage IV	9 (2.2%)	15 (3.6%)	
**Tumor tissue site, n (%)**			0.276
Extremities	104 (24.8%)	93 (22.2%)	
Trunk	97 (23.2%)	74 (17.7%)	
Head and Neck	16 (3.8%)	22 (5.3%)	
Other	5 (1.2%)	8 (1.9%)	
**Melanoma ulceration, n (%)**			< 0.001
No	65 (20.7%)	82 (26.1%)	
Yes	107 (34.1%)	60 (19.1%)	
**Melanoma Clark level, n (%)**			0.020
I	4 (1.2%)	2 (0.6%)	
II	4 (1.2%)	14 (4.3%)	
III	33 (10.2%)	44 (13.7%)	
IV	93 (28.9%)	75 (23.3%)	
V	32 (9.9%)	21 (6.5%)	
**Breslow depth, n (%)**			0.002
≤ 3	83 (23.1%)	102 (28.3%)	
> 3	108 (30%)	67 (18.6%)	
Age, median (IQR)	59 (48, 71)	58 (47, 68)	0.186

**Table 3:** C1QB expressions in clinical features.

Characteristic	Low expression of C1QB	High expression of C1QB	p
n	235	236	
**Gender, n (%)**			0.469
Female	85 (18%)	94 (20%)	
Male	150 (31.8%)	142 (30.1%)	
**Age, n (%)**			0.321
≤ 60	121 (26.1%)	131 (28.3%)	
> 60	112 (24.2%)	99 (21.4%)	
**Race, n (%)**			0.320
Asian	8 (1.7%)	4 (0.9%)	
Black or African American	0 (0%)	1 (0.2%)	
White	222 (48.2%)	226 (49%)	
**T stage, n (%)**			0.003
T1	14 (3.8%)	27 (7.4%)	
T2	42 (11.5%)	37 (10.2%)	
T3	42 (11.5%)	49 (13.5%)	
T4	97 (26.6%)	56 (15.4%)	
**N stage, n (%)**			0.527
N0	120 (29%)	115 (27.8%)	
N1	40 (9.7%)	34 (8.2%)	
N2	21 (5.1%)	28 (6.8%)	
N3	25 (6%)	31 (7.5%)	
**M stage, n (%)**			0.689
M0	210 (47.4%)	208 (47%)	
M1	11 (2.5%)	14 (3.2%)	
**Pathologic stage, n (%)**			0.003
Stage I	29 (7%)	48 (11.7%)	
Stage II	87 (21.1%)	53 (12.9%)	
Stage III	82 (19.9%)	89 (21.6%)	
Stage IV	10 (2.4%)	14 (3.4%)	
**Tumor tissue site, n (%)**			0.419
Extremities	104 (24.8%)	93 (22.2%)	
Trunk	96 (22.9%)	75 (17.9%)	
Head and Neck	17 (4.1%)	21 (5%)	
Other	5 (1.2%)	8 (1.9%)	
**Melanoma ulceration, n (%)**			0.003
No	65 (20.7%)	82 (26.1%)	
Yes	103 (32.8%)	64 (20.4%)	
**Melanoma Clark level, n (%)**			0.032
I	3 (0.9%)	3 (0.9%)	
II	4 (1.2%)	14 (4.3%)	
III	36 (11.2%)	41 (12.7%)	
IV	90 (28%)	78 (24.2%)	
V	34 (10.6%)	19 (5.9%)	
**Breslow depth, n (%)**			0.006
≤ 3	84 (23.3%)	101 (28.1%)	
> 3	106 (29.4%)	69 (19.2%)	
Age, median (IQR)	60 (48, 71)	57.5 (47, 68)	0.129

### Specific pathways of *C1QA*, *C1QB*, and *GZMB*

**Figure 6:**
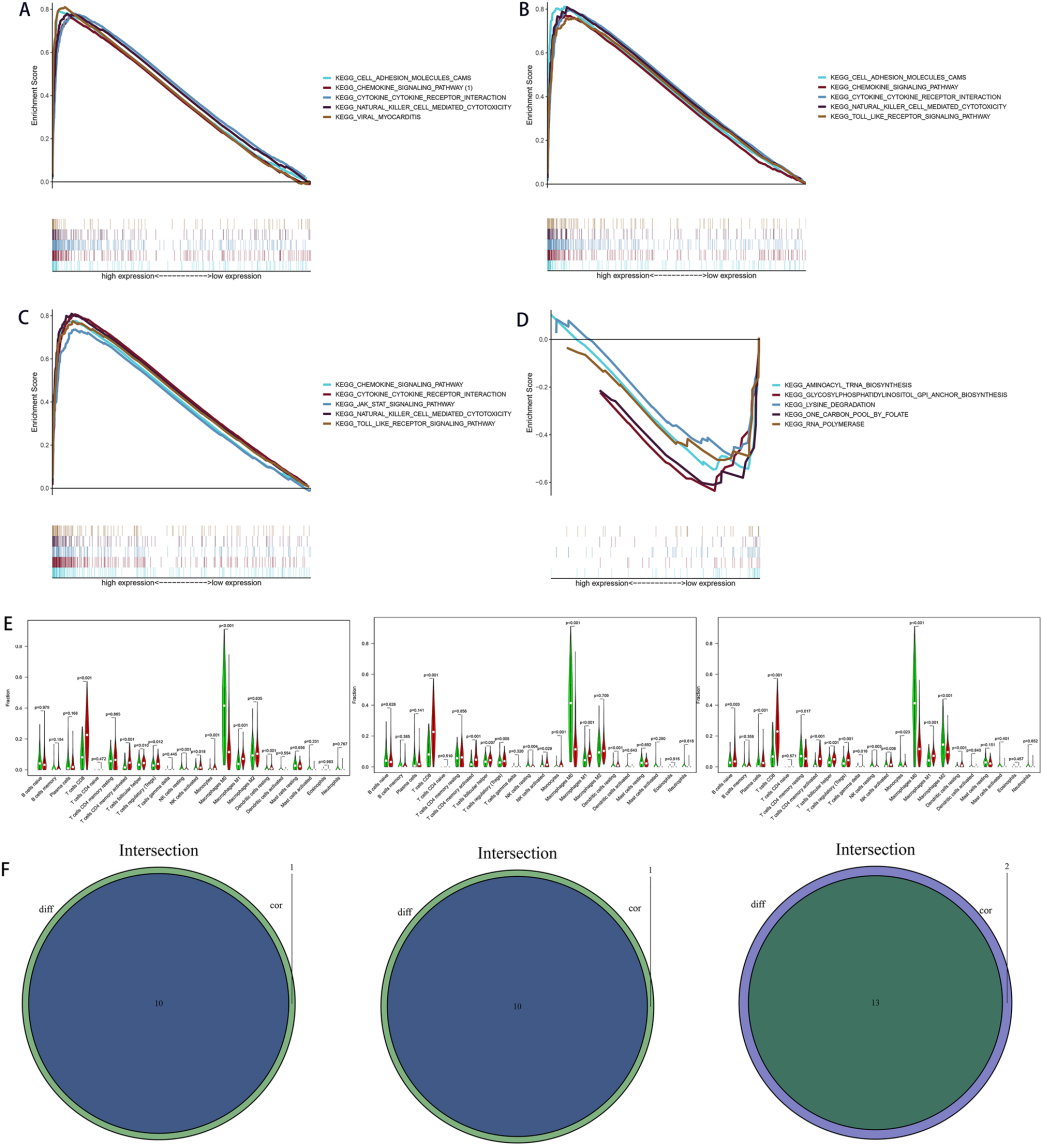
Specific pathways identified by GSEA on the basis of expression levels of *GZMB*, *C1QA*, and *C1QB* and the analyses of associations of *C1QA*, *C1QB* and *GZMB* expressions with TICs of SKCM samples. The top five specific pathways associated with high *GZMB* expressions (**A**), high *C1QA* expressions (**B**), high *C1QB* expressions (**C**) (*P* < 0.05). (**D**) The top five specific pathways associated with low *GZMB* expressions (*P* < 0.05). (**E**) Violin plots of the proportions of 22 immune cell types in tumor tissues with low (green) or high (red) expression of *C1QA*, *C1QB* and *GZMB*, compared using the Wilcoxon rank sum test. (**F**) Venn diagram of intersection between variance analyses and correlation analyses showing that the TICs were shared between the analyses. This figure was generated using the GSEA v4.1.0 software and “ggplot2” R packages.

Because KEGG analysis reveals functional enrichment pathways on the basis of gene category, rather than expression levels of genes, we next used GSEA to identify specific pathways of *C1QA*, *C1QB*, and *GZMB* on the basis of expression levels of the three genes. The expressions of *C1QA* correlated with that of *C1QB* positively (Fig. 5A); thus, we intersected the specific pathways between *C1QA* and *C1QB*. We intersected between the top five specific pathways correlating with high *C1QA* expressions and the top five specific pathways correlating with high *C1QB* expressions, obtaining four intersected specific pathways (chemokine_signaling_pathway, cytokine_cytokine_receptor_interaction, natural_killer_cell_mediated_cytotoxicity, and toll_like_receptor_signaling_pathway) (Fig. 6B,C). However, we did not identify specific pathways shared by both low *C1QA* and *C1QB* expressions. Additionally, the top five specific pathways correlating with high *GZMB* expressions were kegg_chemokine_signaling_pathway, kegg_natural_killer_cell_mediated_cytotoxicity, kegg_viral_myocarditis, kegg_cytokine_cytokine_receptor_interaction, and kegg_cell_adhesion_molecules_cams (Fig. 6A). The top five specific pathways correlating with low *GZMB* expressions were kegg_glycosylphosphatidylinositol_gpi_anchor_biosynthesis, kegg_one_carbon_pool_by_folate, kegg_lysine_degradation, kegg_rna_polymerase, and kegg_aminoacyl_trna_biosynthesis (Fig. 6D). Thus, cell–cell interaction, cell behavior, and intracellular signaling transduction may be mainly involved in both *C1QA* and *C1QB* expressions, as well as in high *GZMB* expressions. Interestingly, metabolism of phospholipid and amino acid, transcription, and translation may be implicated in low *GZMB* expressions.

### The associations of *C1QA*, *C1QB* and *GZMB* expressions with TICs

The analyses of associations of *C1QA*, *C1QB* and *GZMB* expressions with TICs of SKCM samples from TCGA dataset were performed using CIBERSORT algorithm to further investigate the interaction between these genes expression and the TME. The intersections of variance analyses and correlation analyses documented that 10 of 22 TICs were significant related to *C1QA* expression as well as *C1QB* expression, and 13 of 22 TICs were significant related to *GZMB* expression. Interestingly, most of these TICs such as CD 4 and CD 8 T cells, M1 and M2 macrophages were positive correlated to the expression of these three genes. thus conferring a significant survival advantage (Fig. 6E,F) (Fig. S2). These results suggested that *C1QA*, *C1QB* and *GZMB* play vital roles in immune infiltration processes in SKCM patients and represented potential therapeutic targets.

### The correlations between *C1QA*, *C1QB *and *GZMB*

**Figure 7:**
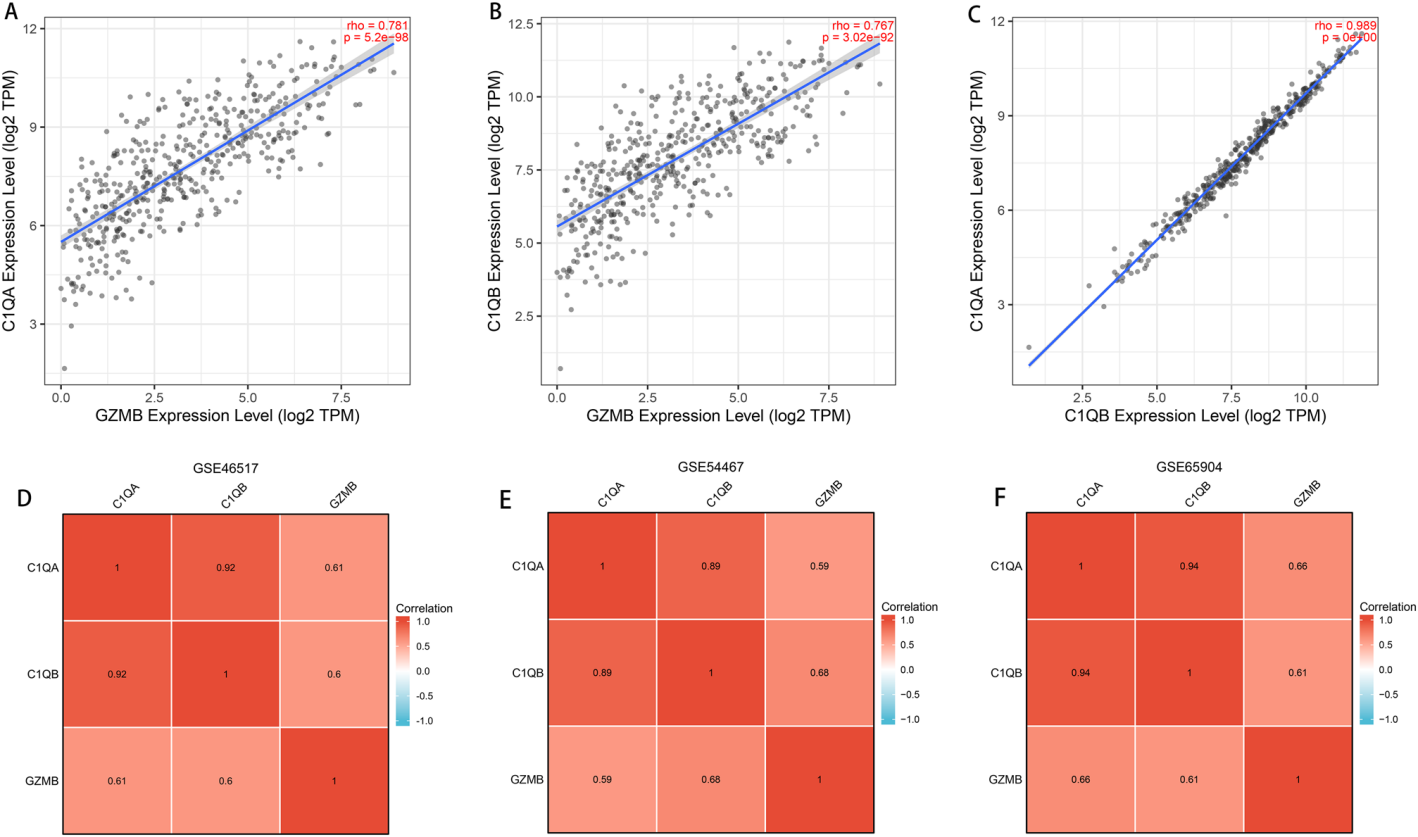
The correlations between expressions of *C1QA*, expressions of *C1QB* and expressions of *GZMB* in (**A**–**C**) TCGA, (**D**) GSE46517, (**E**) GSE54467, and (**F**) GSE65904. This figure was generated using the TIMER2.0 web tool (http://timer.comp-genomics.org/)^30^ and “ggplot2” R package.

We investigated the correlations between expressions of *C1QA*, expressions of *C1QB* and expressions of *GZMB.* Interestingly, we found that the expressions of *C1QA* positively correlated with those of *C1QB* and *GZMB,* and expressions of *C1QB* positively correlated with those of *GZMB*, which indicated that *C1QA*, *C1QB* and *GZMB* function as synergistic roles in the developments of SKCM (Fig. 7).

### Single-cell analysis of *C1QA*, *C1QB *and *GZMB* in SKCM

**Figure 8:**
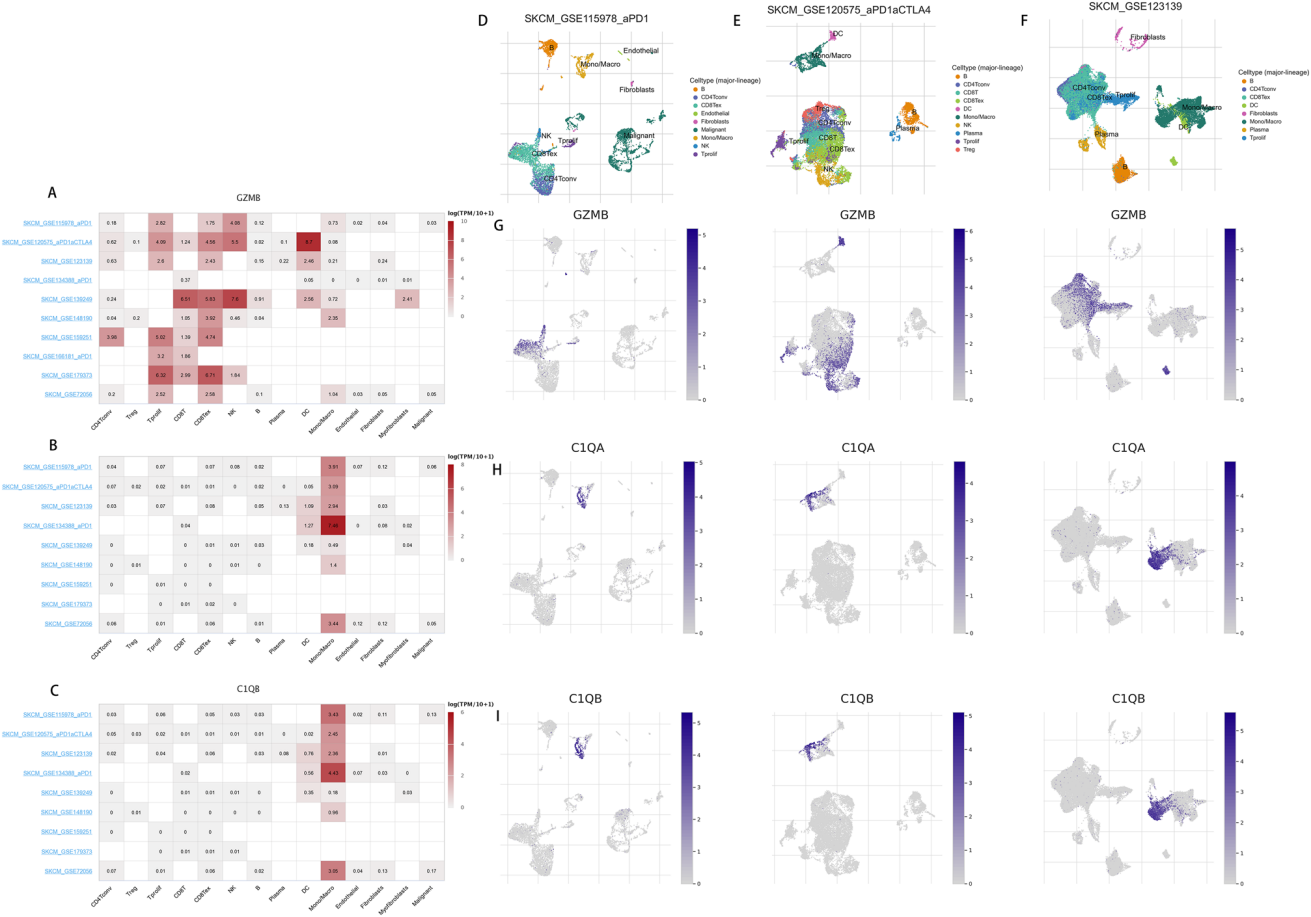
(**A**–**C**) Summary of *C1QA*, *C1QB* and *GZMB* expressions of 14 cell types in 9 SKCM single cell datasets. (D-F) Scatter plot showed the distributions of different cell types of the SKCM single cell dataset. (**G**–**I**) Scatter plot showed the *C1QA*, *C1QB* and *GZMB* expressions levels of cells in the SKCM single cell dataset. This figure was generated using the Tumor Immune Single-cell Hub (TISCH) web tool (http://tisch.comp-genomics.org/documentation/)^18^.

We further understand the main cell types in SKCM microenvironments that express the *C1QA*, *C1QB* and *GZMB*. The heatmaps showed that GZMB was mainly enriched in the immune cells (especially T cells), while *C1QA*, *C1QB* were mainly expressed in monocyte/macrophage (Fig. 8).

## Discussion

In this study, we investigated TME-related genes involved in SKCM, indicating that the six CTRGs (*CCL4*, *CXCL10*, *CCL5*, *GZMB*, *C1QA*, and *C1QB*) affect OS in patients with SKCM. Our results also support the link of melanoma with *CCL4*, *CXCL10*, and *CCL5*^10^. We verified *GZMB*, *C1QA*, and *C1QB* as novel SKCM-relating CTRGs.

*CCL4*, *CCL5*, and *CXCL10* have been reported as potential biomarkers for SKCM on May 25th, 2020^10^. As expected, the expressions of *CXCL10*, *CCL4* and *CCL5* in SKCM are significantly higher than those in normal tissues. Moreover, *CCL5* modulates tumor immune responses via a local renin-angiotensin system in malignant melanoma^21^. In addition, the low transcription levels of *CXCL10* correlate with better prognosis in patients with SKCM^22^.

We identified three novel SKCM-relating CTRGs, namely *C1QA*, *C1QB*, and *GZMB*. Complement 1 is composed of C1q, C1r, and C1s. C1q recognizes and binds to immunoglobulin complexed to antigen and initiates the complement cascade. *C1QA* encodes the A-chain polypeptide of serum complement subcomponent C1q (C1QA), and *C1QB* encodes the B-chain polypeptide of serum complement subcomponent C1q (C1QB). Both C1QA and C1QB are implicated in complement pathway and innate immune system. Additionally, *C1QA* and *C1QB* have been found as indices of TME remodeling in osteosarcoma^23^. Granzyme B encoded by *GZMB* is secreted by natural killer cells and cytotoxic T lymphocytes, proteolytically processing cytokines and degrading extracellular matrix proteins. Granzyme B is involved in apoptosis by cleaving caspase-3, -7, -9 and -10^24^. Moreover, granzyme B triggers caspase-independent pyroptosis after delivered into the target cell via the immunological synapse^25–27^. Indeed, pyroptosis-related gene signatures have been showed in robustly predicting the prognosis of SKCM^28^. Of note, CD8+ T cells propagates autoimmunity via granzyme-B-generated unique autoantigen fragments; whereas, C1q limits tissue damage and autoimmunity by mediating effector CD8+ T cells, providing biological insight into an interconnectivity between C1q and granzyme B^29^. We found that expression levels of *GZMB*, *C1QA*, and *C1QB* in normal samples of TCGA dataset were significantly reduced, compared with those in SKCM samples. Thus, the interconnectivity between C1q and granzyme B may recapitulate the three novel SKCM-relating CTRGs (*C1QA*, *C1QB*, and *GZMB*) as biomarkers for the occurrence and development of SKCM.

There are limitations in this study. Firstly, *C1QC* encodes the C-chain polypeptide of serum complement subcomponent C1q; however, we did not know the reason why there is no correlation of *C1QC* with SKCM. Secondly, we used sequencing data sets from multiple databases to investigate SKCM-relating CTRGs. Multi-omic data are needed to be further validate our results.

In conclusion, *C1QA*, *C1QB*, and *GZMB* are novel SKCM-relating CTRGs, providing promising immune-related prognostic biomarkers for SKCM.

## Conclusion

*C1QA*, *C1QB*, and *GZMB* are novel SKCM-relating CTRGs, providing promising immune-related prognostic biomarkers for SKCM.

## Supplementary Information

Supplementary Figures.

## Data Availability

The datasets used during the current study are available from the corresponding author on reasonable request.
