# Publisher Correction: *C1QA*, *C1QB*, and *GZMB* are novel prognostic biomarkers of skin cutaneous melanoma relating tumor microenvironment

**DOI:** 10.1038/s41598-023-37876-6

**Published:** 2023-07-06

**Authors:** Zhuoshuai Liang, Lingfeng Pan, Jikang Shi, Lianbo Zhang

**Affiliations:** 1 Department of Epidemiology and Biostatistics, School of Public Health, Jilin University, Changchun, 130021 Jilin China; 2 Department of Plastic Surgery, China Japan Union Hospital of Jilin University, Changchun, 130033 Jilin China

Correction to: *Scientific Reports* 10.1038/s41598-022-24353-9, published online 28 November 2022

The original version of this Article contained an error in the Funding section.

“This work was supported by the funds from the National Natural Science Foundation of China (No. 81971842 to Lianbo Zhang) (grant numbers 81973120 to Liu) and National Key R&D Program of China (grant numbers #2018YFC1311600 to Liu).”

now reads:

“This work was supported by the funds from the National Natural Science Foundation of China (No. 81971842 to Lianbo Zhang).”

The original Article has been corrected.

