# Supplementary material for: C1QA, C1QB, and GZMB are novel prognostic biomarkers of skin cutaneous melanoma relating tumor microenvironment
Source: Sci Rep. 2022 Nov 28;12:20460. doi: 10.1038/s41598-022-24353-9 (PMC9705312; doi:10.1038/s41598-022-24353-9)

Figure S1. Forest plot of univariate Cox regression analyses, showing genes with P < 0.001.


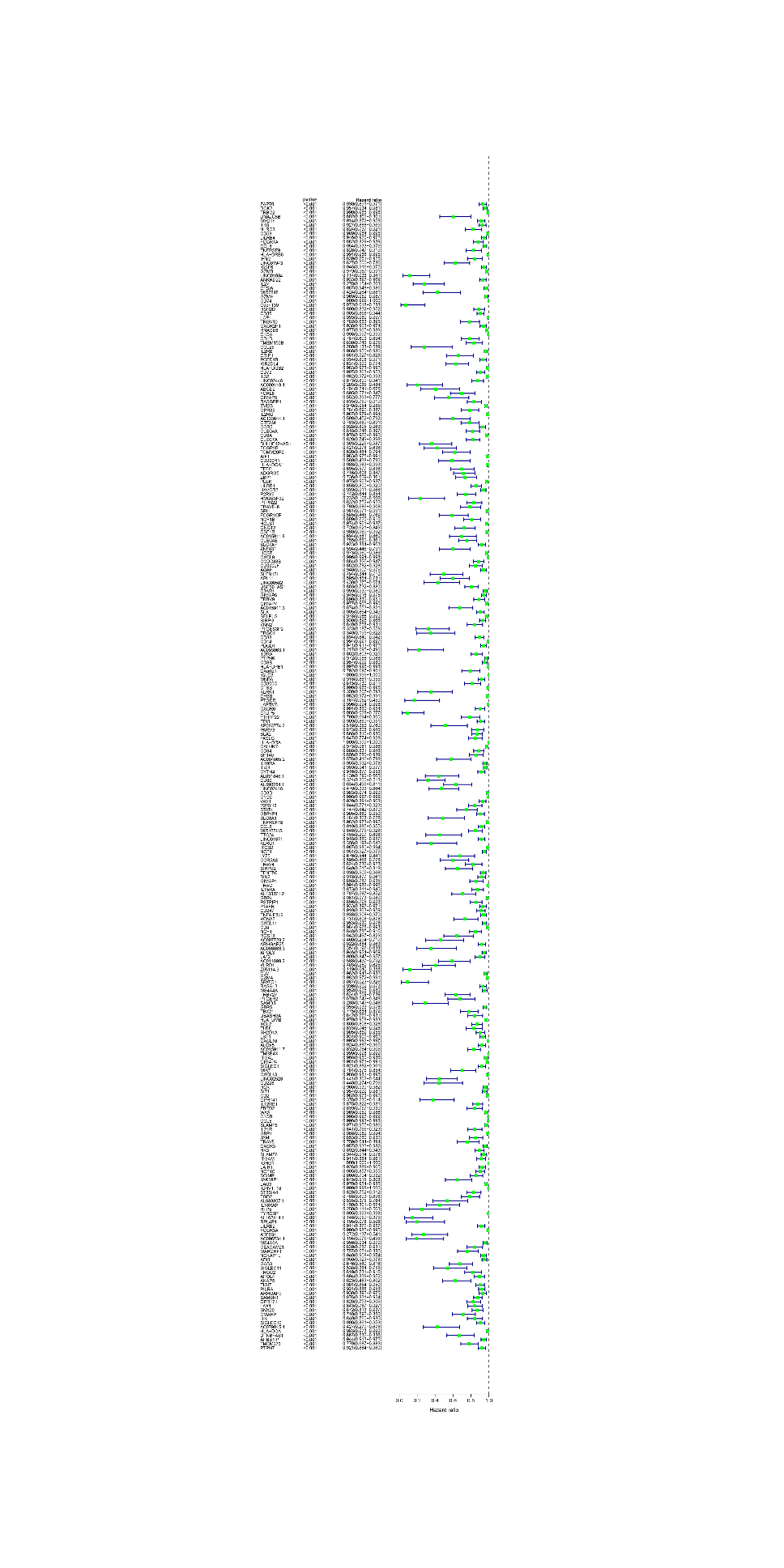


Figure S2. Scatter plots showing Pearson’s correlation between the proportions of the most significant TICs and the expression of C1QA (A), C1QB (B) and GZMB (C). Blue lines denote the best-fit linear models.


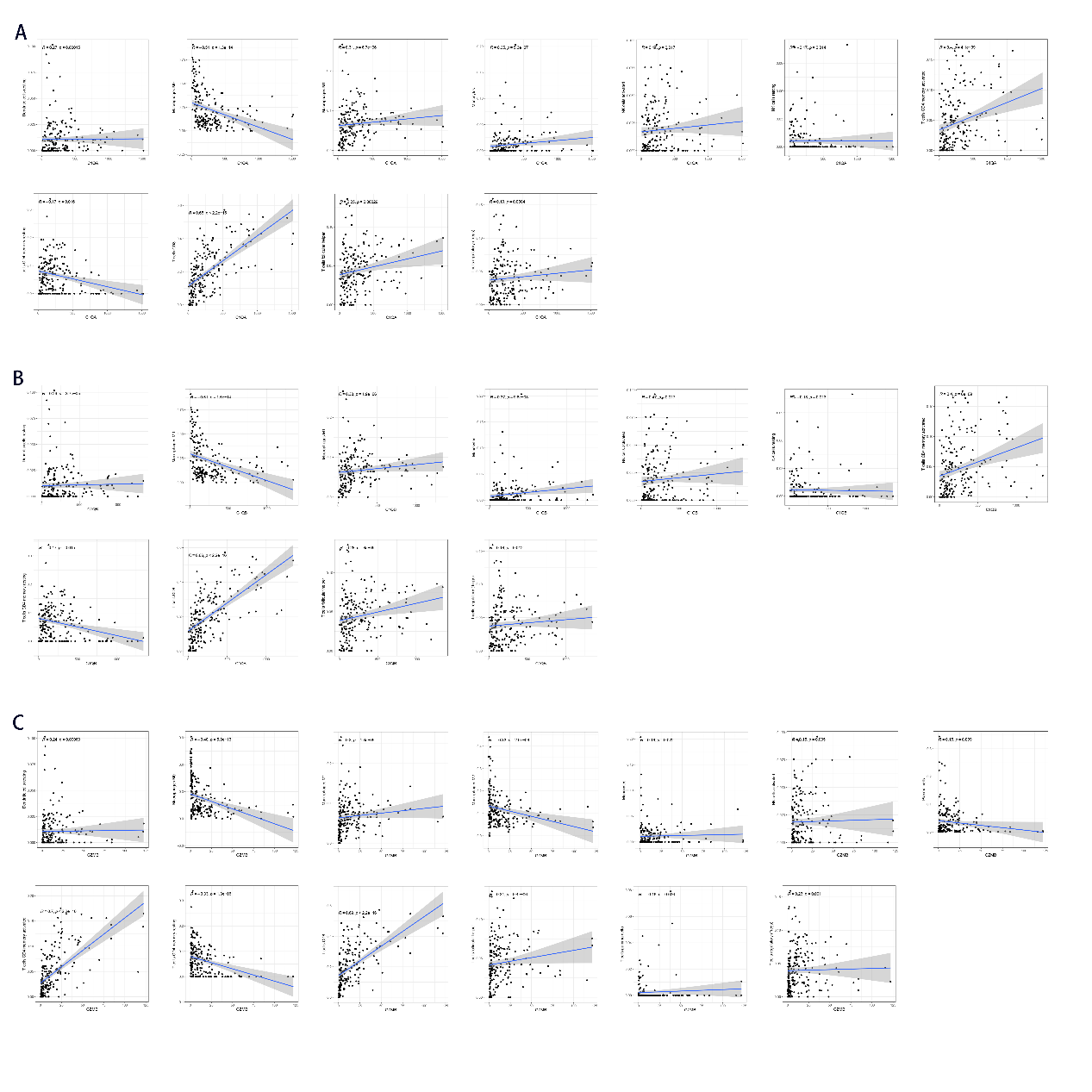

Supplement: Supplementary file 1 — Supplementary Figures. [file 41598_2022_24353_MOESM1_ESM.docx]
